# The spatiotemporal estimation of the risk and the international transmission of COVID-19: a global perspective

**DOI:** 10.1038/s41598-020-77242-4

**Published:** 2020-11-18

**Authors:** Yuan-Chien Lin, Wan-Ju Chi, Yu-Ting Lin, Chun-Yeh Lai

**Affiliations:** 1 Department of Civil Engineering, National Central University, Taoyuan, 32001 Taiwan; 2 Research Center for Hazard Mitigation and Prevention, National Central University, Taoyuan, 32001 Taiwan

**Keywords:** Infectious diseases, Public health

## Abstract

An ongoing novel coronavirus outbreak (COVID-19) started in Wuhan, China, in December 2019. Currently, the spatiotemporal epidemic transmission, prediction, and risk are insufficient for COVID-19 but we urgently need relevant information globally. We have developed a novel two-stage simulation model to simulate the spatiotemporal changes in the number of cases and estimate the future worldwide risk. Simulation results show that if there is no specific medicine for it, it will form a global pandemic. Taiwan, South Korea, Hong Kong, Japan, Thailand, and the United States are the most vulnerable. The relationship between each country's vulnerability and days before the first imported case occurred shows an exponential decrease. We successfully predicted the outbreak of South Korea, Japan, and Italy in the early stages of the global pandemic based on the information before February 12, 2020. The development of the epidemic is now earlier than we expected. However, the trend of spread is similar to our estimation.

## Introduction

In December 2019, a novel coronavirus SARS-CoV-2 infection pneumonia outbreak COVID-19 (2019-nCoV) started in Wuhan, Hubei Province, China^1–3^. It spread rapidly to all provinces throughout China and started spreading around the world quickly through international human movement from January 2020^2,4^. Thus far (February 12, 2020), the epidemic has not decelerated but has started breaking out internationally. For example, cluster outbreaks have occurred in Singapore, Japan, and on international cruise ships (e.g. the Diamond Princess). With the exception of China, countries are at the most critical stage to avoid outbreaks of domestic cluster infections.

Many studies or reports have started revealing that SARS-CoV-2 is very infectious^2,5^. Many cases are asymptomatic or mildly diagnosed patients^6^, which greatly increases the potential for transmission and makes epidemic prevention very difficult. Therefore, it will likely evolve into a global pandemic in the future.

Many countries have started implementing measures to prevent the epidemic spreading, including stopping flights from China, quarantining at airports, suspending visas for Chinese citizens, issuing travel warnings to China and advising citizens not to visit China unless necessary, and implementing home quarantine or stopping the entry and transfer of foreign tourists who have traveled to China within 14 days to prevent imported cases after overseas traveling. However, since many countries have very close business or human relationships with China, it is impossible to avoid imported cases. Therefore, to understand the initial transmission of novel infectious disease, we must first understand each country’s connectivity to the country of origin, which is the first step for countries around the world to prevent the first stage of overseas imported cases.

Since the outbreak, we all want to know how COVID-19 will spread from Wuhan all across China and further from China to all around the world in terms of both time and space. A previous study made preliminary estimations and predictions through a typical infectious disease model called a susceptible–exposed–infectious–recovered (SEIR) model^5^; however, as it is a new infectious disease, it is difficult for us to understand how the epidemic will develop in the future such as the parameters when the peak is reached. Unknown infectious diseases have made it very difficult to adjust and calibrate the parameters of various prediction models like the SEIR model, making the model predictions very uncertain. The totally different pattern of transmission means that all spatiotemporal models or patterns from other coronaviruses such as the 2003 severe acute respiratory syndrome (SARS) outbreak and the Middle East respiratory syndrome (MERS) are less suitable for use with COVID-19, even if they have highly similar genome sequence identity^7,8^.

Currently, the spatiotemporal epidemic transmission patterns, prediction models, and possible risk analyses for the future at the global-scale remain insufficient for COVID-19 but we urgently need relevant information. This is an important and critical time for global early-stage epidemic control and public health issues. It is difficult for us to predict future case changes in various countries, particularly the first stage of overseas imported cases from China and the second stage of local transmission cases. Under this background of high uncertainty around COVID-19, our study aims at global COVID-19 risk analysis from a data analysis perspective.

**Figure 1:**
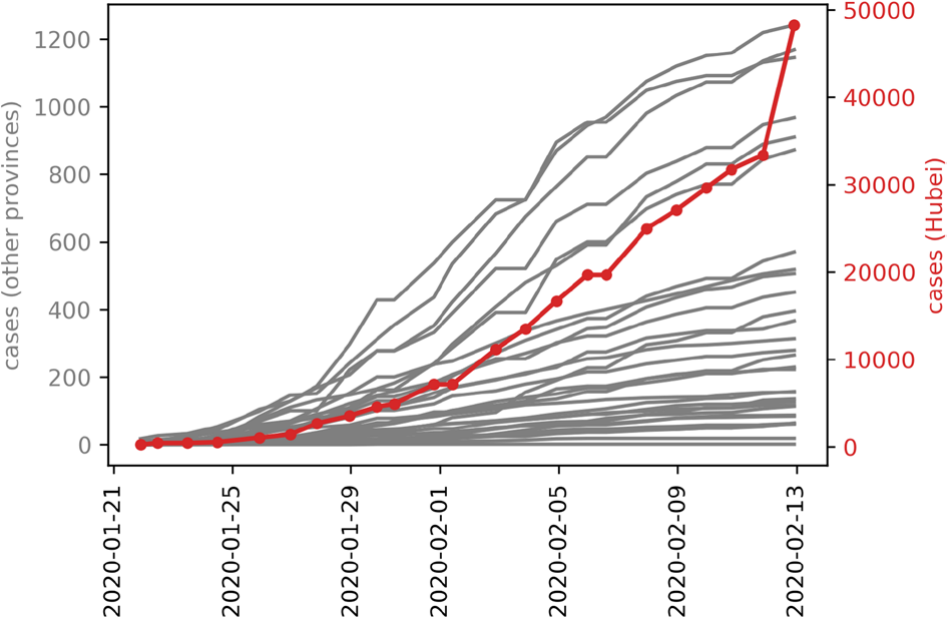
The cumulated confirmed case numbers of all provinces in China for 2020/1/21–2020/2/12 (red: Hubei, gray: all other provinces).

Understanding the connectivity with the country of origin for infectious diseases, i.e. China, is a useful way for countries to quantitatively evaluate the risk of imported cases. Higher connectivity is indicative of a higher risk of importation. In addition, for the analysis of worldwide countries other than China, we can use the changes in the number of cases that have been widespread in China's provinces to estimate the number of cases worldwide. Based on the comparison of the actual confirmed cases between Hubei and the average from other provinces, other provinces are at least 15 days late in Hubei Province on average to reach a similar number of cases. In other words, except for Hubei Province, China, where Wuhan is located, the epidemic pattern of China’s provinces precedes that of countries around the world by about 0.5–1 months. Thus, this can be used as a dynamic reference basis for case prediction, simulation, and risk analysis in various countries (Fig. 1).

In this study, we developed a novel two-stage simulation model to simulate the spatiotemporal changes in the number of COVID-19 cases and estimate the future worldwide risk based on a data-driven approach. Based on each country’s connectivity to China, medical and epidemic prevention capabilities, and future conditions, different scenarios are generated with which to analyze the potential transmission around the world. Meanwhile, from a risk management perspective, understanding risk and proper risk management is a very important part of preventing the spread of infectious diseases in various countries. To assess the risk of COVID-19 from China, we first defined vulnerability as the country's inability to resist to coronavirus outbreaks based on the Health Care Index and the connectivity to the outbreak country. It is defined as the summation of connectivity divided by the Health Care Index for each country. The more the connectivity to the outbreak countries, the higher the vulnerability, on the other hand, the more health care ability will reduce the vulnerability. Secondly, the exposure is seen as potentially affected by the domestic population of each country. Lastly, the degree of hazard means how harmful is the disease to the countries, that decided by the number of infected cases. After calculating each country's exposure, the degree of hazard, and vulnerability, we generated comprehensive spatiotemporal dynamic risk maps of COVID-19 over time.

## Result

- Calibration of China’s confirmed case number

Since the local medical system in Hubei Province may be insufficient to support the huge number of patients, the official diagnoses may be an underestimate. Therefore, we first collected information on the evacuation flights from different countries at different times to calculate the proportion of confirmed cases and used this to estimate the infection rate in Wuhan based on the concept of statistical sampling, as shown in Table 1.

**Table 1:** The confirmed cases and number of evacuation flights ( *Source*: Collected from the international press).

Date	Country	Number of people on flights	Confirmed cases	Suspected cases	Confirmed cases after quarantine	Proportion of confirmed cases (%)	Remark
2020/1/29	Japan	206	3		1	1.94	Two asymptomatic cases
2020/1/29	United States	195	0			0.00	
2020/1/30	Japan	210	2			0.95	
2020/1/31	Japan	149	3		1	2.68	
2020/1/31	France	180	0	1		0.00	
2020/2/1	Germany	124	2			1.61	
2020/2/2	France	254	0	36		0.00	The test for suspected cases are all negative
2020/2/2	Indonesia	237	0			0.00	
2020/2/3	Malaysia	141	2			1.42	
2020/2/3	Australia	243	0			0.00	
2020/2/3	Vietnam	194	0			0.00	
2020/2/4	Taiwan	247	1	3		0.40	Confirmed patients and fever patients are not allowed to board aircraft
2020/2/4	Thailand	138	0		1	0.72	Two of the original 141 people did not board the plane because of fever
2020/2/5	United States	350	0			0.00	
2020/2/5	Russia	80	0			0.00	
2020/2/6	United States	410	0		1	0.00	
2020/2/7	Japan	198	1	4		0.51	
SUM_1_		3556	14	44	4	0.51	
SUM_2_ (Japan, Germany, Malaysia)		1028	13	4	2	1.46	

Sample 1 was calculated based on the average of all cases in the countries collected in Table 1. The proportion P_1_ = 0.51%, with 95% confidence interval [0.32%, 0.80%]. There are nine million current residents in Wuhan as announced by the press conference held by the Hubei Provincial Government of China on the evening of 1/26; of these, approximately 45,900 people with 95% confidence interval [28,800, 72,000] may be infected. As of the official announcement of 2020/2/8, the number of confirmed cases in Hubei has reached 27,100 people, and the number of confirmed cases in China has reached 34,546 people. This estimation sample is insufficient to show the number of hidden cases.

According to the news press and public information, avoiding onboard infection or the triggering of more domestic infections, many countries have screened evacuating people, such as those who have a fever or with confirmed infections, to prevent them from boarding planes. Therefore, this sampling will be underestimated.

Sample 2 only collected cases of Japanese, German, and Malaysian evacuation charter flights as these may be more relevant for sampling. In other words, since this is especially for the evacuation flight, it is still possible to underestimate the overall Hubei cases, so we select some countries that have particular cases in the flight to count the worst scenario. The proportion P_2_ = 1.46%, with 95% confidence interval [0.89%, 2.39%]. Based on the nine million people living in Wuhan, the average number of people infected during early February was about 131,400 with 95% confidence interval [80,100, 215,100]. The confidence interval for a population proportion *π* with a confidence level of 95% is calculated by:

1 $$ \frac{{p + \frac{{z*^{2} }}{2n} \pm z*\frac{{\sqrt {p(1 - p)} }}{n} + \frac{{z*^{2} }}{{4n^{2} }}}}{{1 + \frac{{z*^{2} }}{n}}} $$

where z* denotes an appropriate critical value, here we use 0.95 for 95% confidence interval; *p* is the sample proportion; *n* is the sample size^9^.

b. Vulnerability

**Figure 2:**
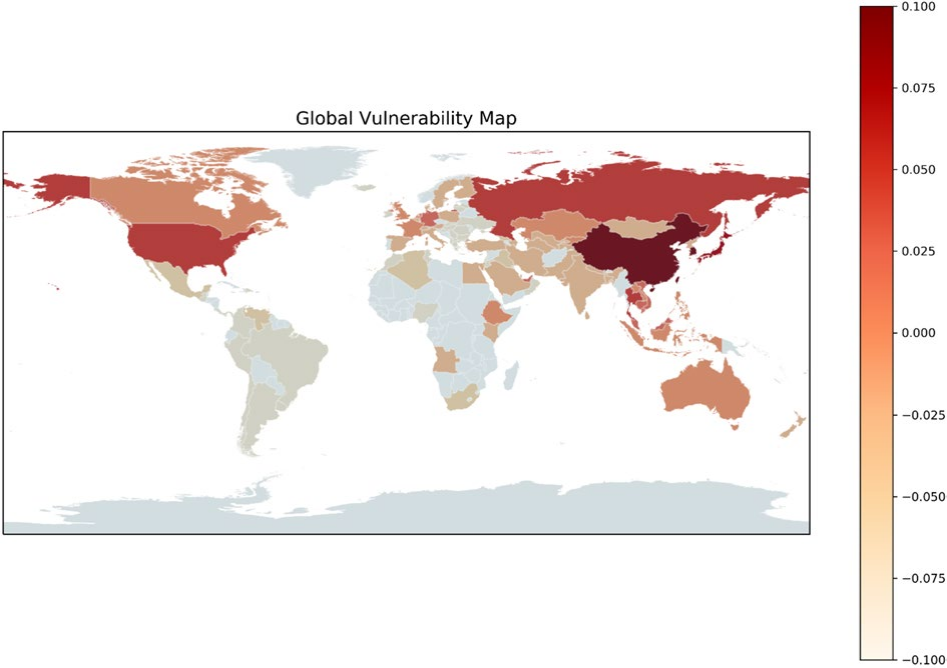
Global vulnerability map for the COVID-19 outbreak from China. The map is generated by YCL in Python 3.7 (https://www.python.org/) through the development environment Anaconda 5.3.0 (https://www.anaconda.com/).

Vulnerability to the COVID-19 outbreak from China is calculated for 63 countries around the world, as shown in Fig. 2. Table 2 shows the first 20 countries’ vulnerability. Taiwan has the highest vulnerability to novel coronavirus outbreak from China, or broadly speaking to various human infectious diseases from China, followed by South Korea, Hong Kong, and Japan. These countries are the most vulnerable, followed by Thailand, the United States, Russia, Macau, and Singapore. The top-20 countries listed in Table 2 are considered relatively vulnerable.

**Table 2:** The first 20 countries’ vulnerability to the COVID-19 outbreak from China (without China).

Rank	Country	Vulnerability
1	Taiwan	1.000
2	South Korea	0.848
3	Hong Kong	0.809
4	Japan	0.791
5	Thailand	0.426
6	United States	0.387
7	Russia	0.373
8	Macau	0.326
9	Singapore	0.287
10	Malaysia	0.194
11	Vietnam	0.182
12	Cambodia	0.120
13	Germany	0.118
14	United Arab Emirates	0.101
15	Australia	0.099
16	Indonesia	0.097
17	Burma	0.091
18	Philippines	0.087
19	Netherlands	0.078
20	Canada	0.075

**Figure 3:**
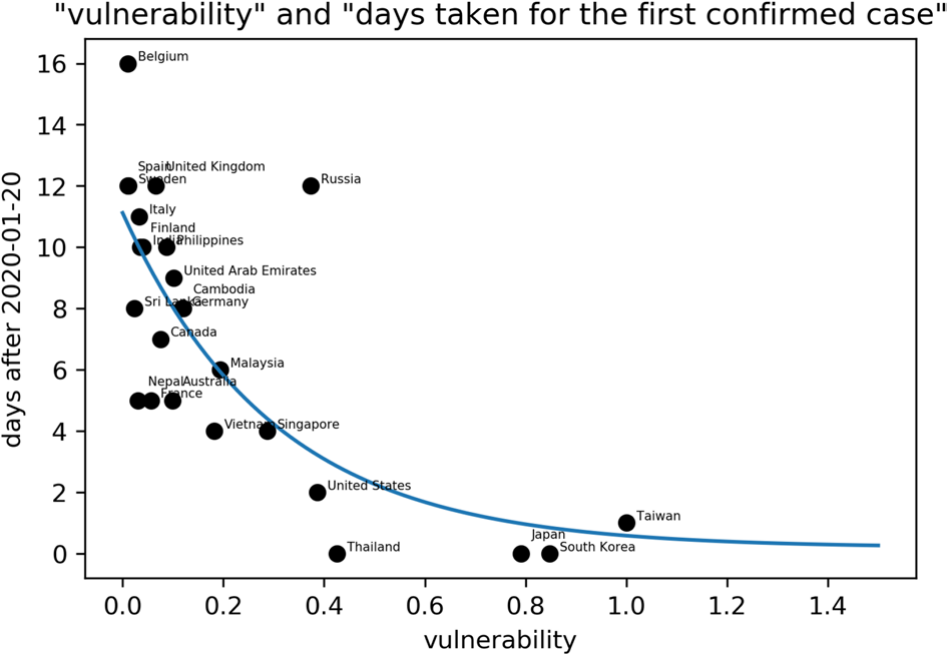
The relationship between each country’s vulnerability and how many days it took before the first imported case occurred. The fitted regression function is *y* = *10.93exp(−3.32x)* + *0.19.*

Without effective anti-epidemic measures in these countries, cases of overseas imports from China may soon occur. Figure 3 observes the relationship between each country’s vulnerability and days taken for the first imported case to occur; they show a very high exponential decreasing relationship. This result proves our argument and confirms that the vulnerability index proposed by this study is very informative and can well express the possibility and number of cases that may happen in the first stage for each country. Although different countries' immediate epidemic prevention policies may affect this; overall, the more vulnerable a country, the sooner its first overseas import case from China would occur. Meanwhile, the lower the vulnerability, the later such a case would occur.

**Figure 4:**
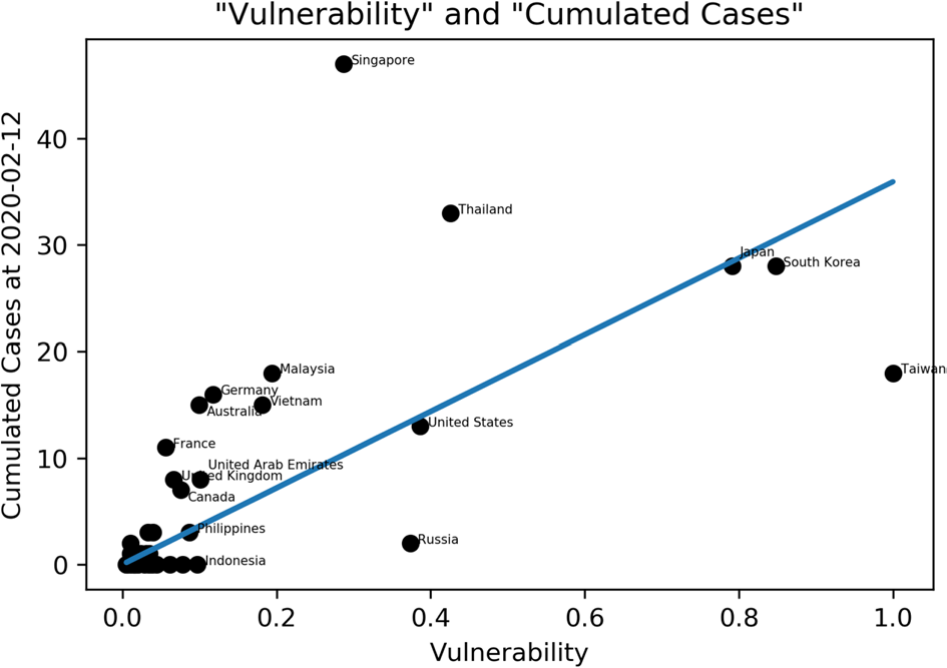
The relationship between vulnerability and the cumulative number of cases in each country before 2020–02-12. The coefficient of the OLS Regression equation is 35.94 with R^2^ = 0.587 and 95% C.I = [26.896, 44.994].

In addition, we can observe the relationship between vulnerability and the cumulative number of cases in each country before 2020-02-12 as shown in Fig. 4; this shows a clear linear relationship. This is a dynamic process that changes daily with the number of cumulative cases. Since the beginning of the outbreak in January 2020, various countries’ governments have implemented related measures for epidemic prevention such as reducing flights and stopping visas so that many countries with high vulnerability can maintain a small number of cases. The countries below this regression line—including Taiwan, Korea Republic, and Russia—can be regarded as countries with excellent “initial” or “early stage” epidemic prevention work and Japan and the United States have performed fairly well (unfortunately, Japan and Korea Republic starting finding outbreak community infections after this study was completed), whereas Singapore and Thailand are far above the regression line with higher cases and may have higher risk in the future. Please noticed that this figure will change with the number of cases in different countries at different times.

c. Simulation result and dynamic risk analysis

**Figure 5:**
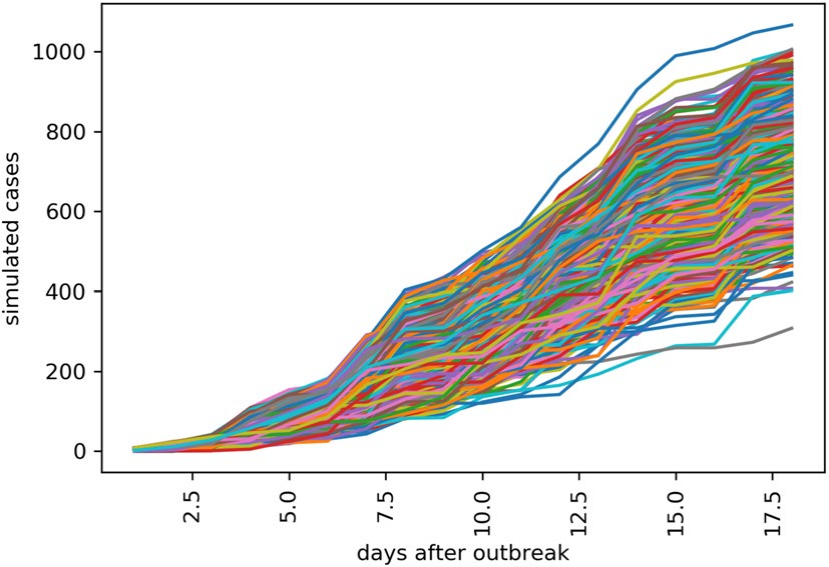
Time series of the 1,000 countries simulated case numbers after the second-stage outbreak in the future using Monte Carlo simulation.

**Figure 6:**
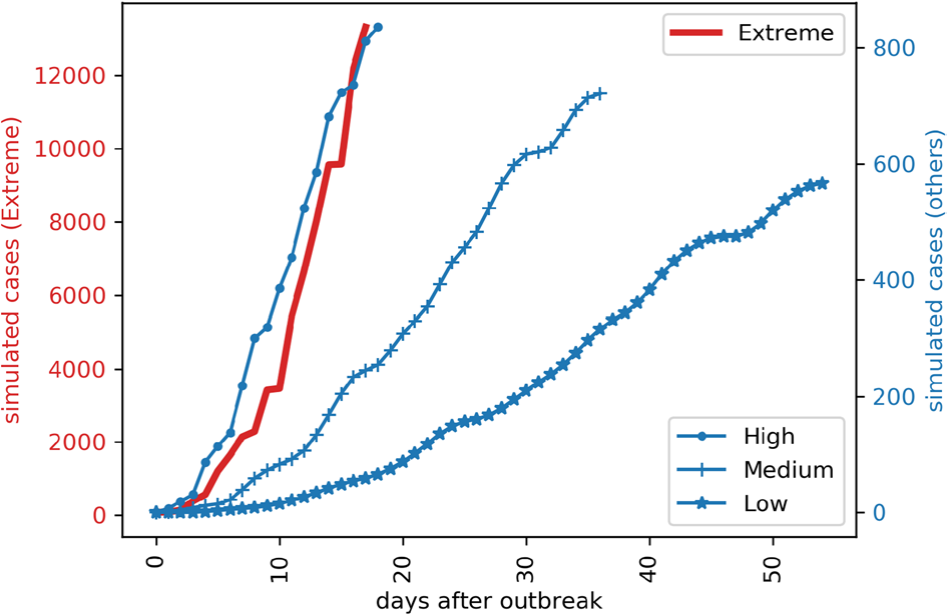
The simulated number of potential cases in four different scenarios: high, medium, low, and the extreme scenarios of Hubei Province.

**Figure 7:**
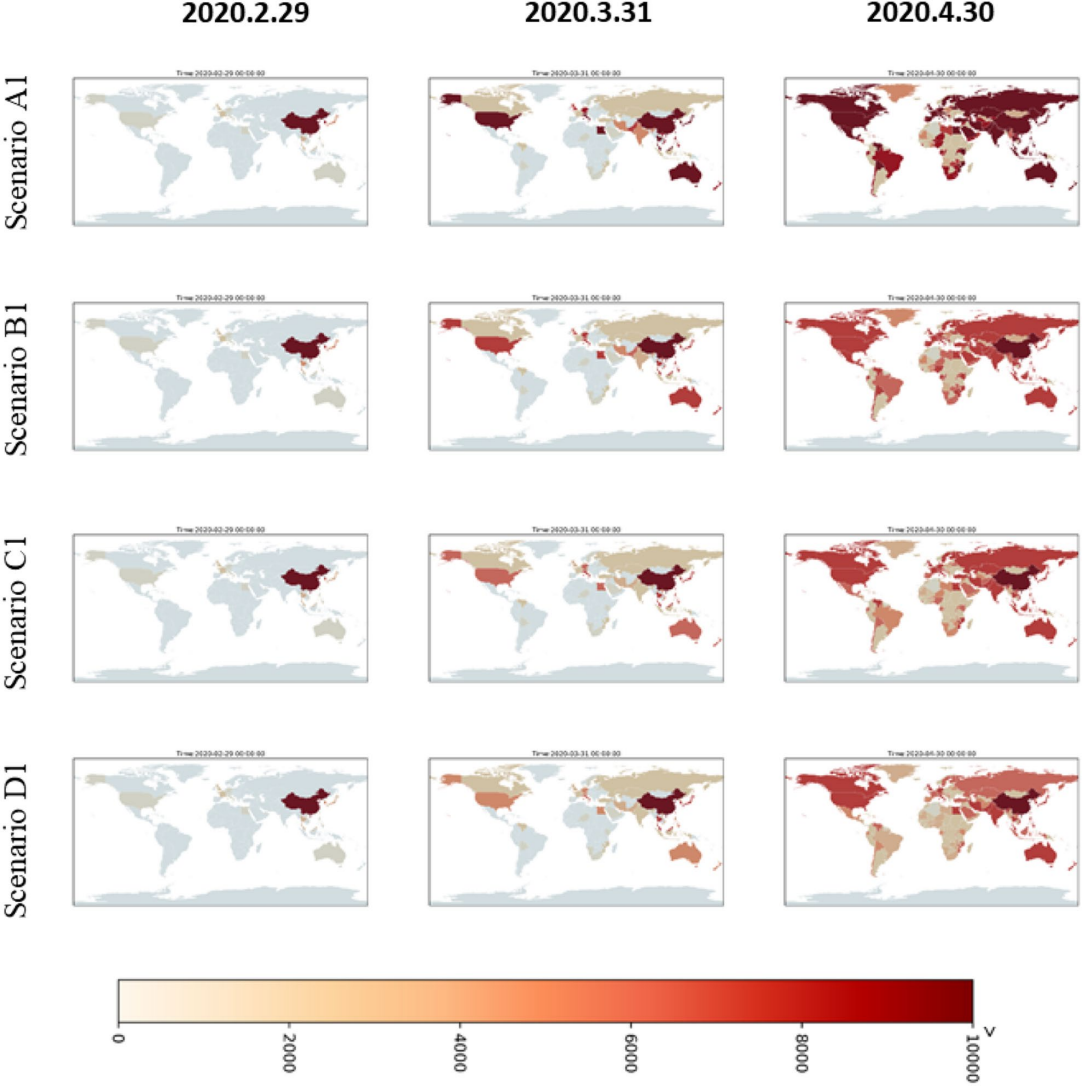
The simulated cumulative case maps for conservative scenarios of A1, B1, C1, and D1 in different time-slices. The maps is generated by YCL in Python 3.7 (https://www.python.org/) through the development environment Anaconda 5.3.0 (https://www.anaconda.com/).

**Figure 8:**
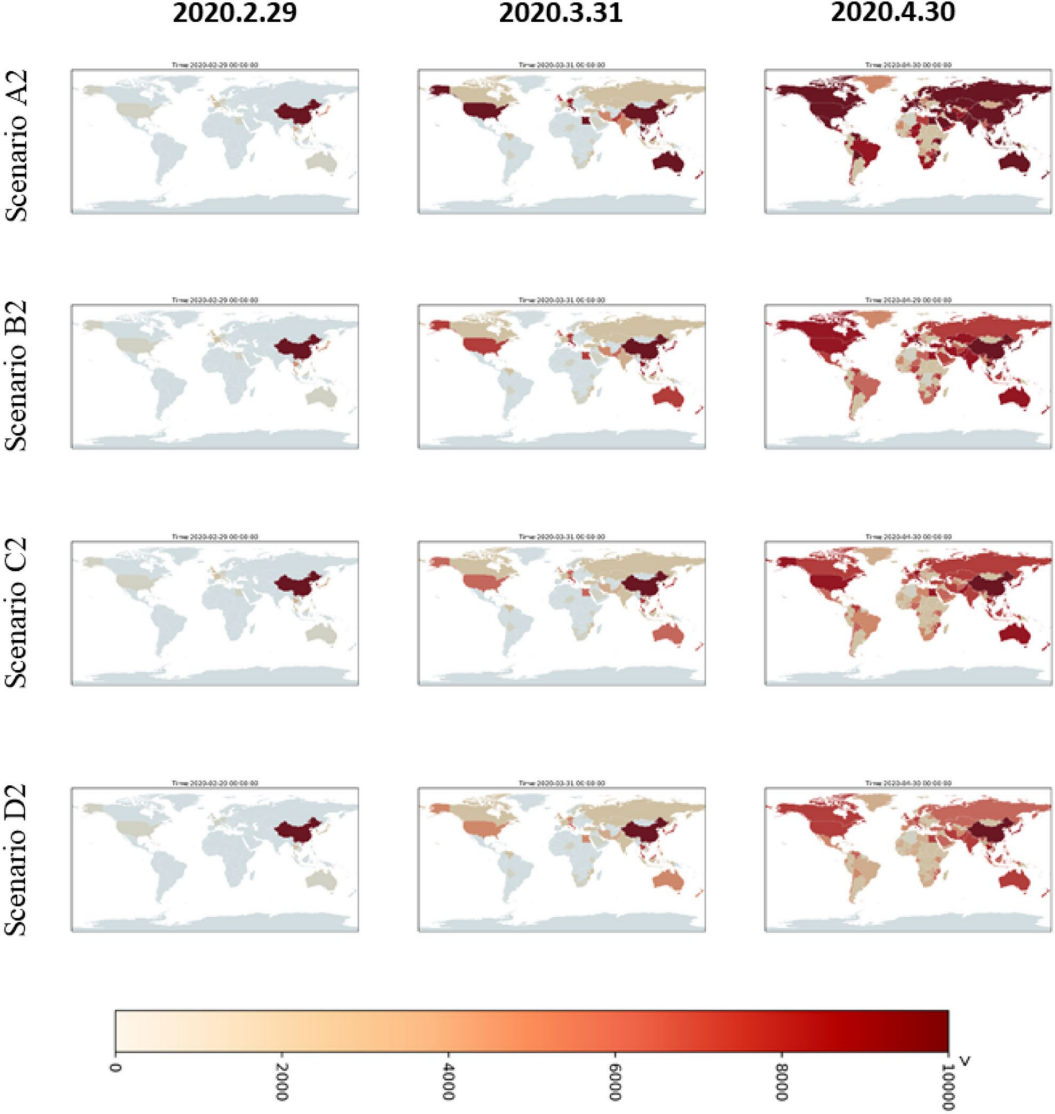
The simulated cumulative case maps for severe scenarios of A2, B2, C2, and D2 in different time-slices. The maps is generated by YCL in Python 3.7 (https://www.python.org/) through the development environment Anaconda 5.3.0 (https://www.anaconda.com/).

Figure 5 is the time series of the 1000 countries’ simulated case numbers after the second stage outbreak in the future using Monte Carlo simulation. Figure 6 is the simulated number of potential cases under four different scenarios: high, medium, and low, and the extreme scenarios of Hubei Province. After combining the simulation results of the countries’ first and second stages around the world using the data fusion algorithm, the simulated cases in different future scenarios are generated as shown in Figs. 7 and 8.

According to the spatiotemporal distribution results of different scenario simulations, the conservative scenarios (Fig. 7) assume that China has entered the start of its peak period and the number of outbreak cases has started to decline. Therefore, the results of simulations applied in other countries show that the cumulated case number increases rapidly at the start for some countries, particularly for countries with higher vulnerability. After one month, the epidemic was controlled and the number of cases in each country no longer increased significantly. Meanwhile, considering the extreme, high, medium, and low simulation levels of the epidemic scenarios in various countries, it can also be seen that after a period of time, namely in April, the case number differences between the scenarios will be clearly comparable. A1 extreme is the highest scenario, followed by B1, C1, and D1. Meanwhile, severe scenarios (Fig. 8) are based on the trend that COVID-19 may be like traditional influenza for a global pandemic; that is, cases will continue to occur, which may lead to > 10,000 cases in some countries. However, the general spatial distribution of case numbers is similar to conservative scenarios. This scenario is still the same as that with the highest case numbers in the A2 extreme scenario, followed by B2, C2, and D2.

**Figure 9:**
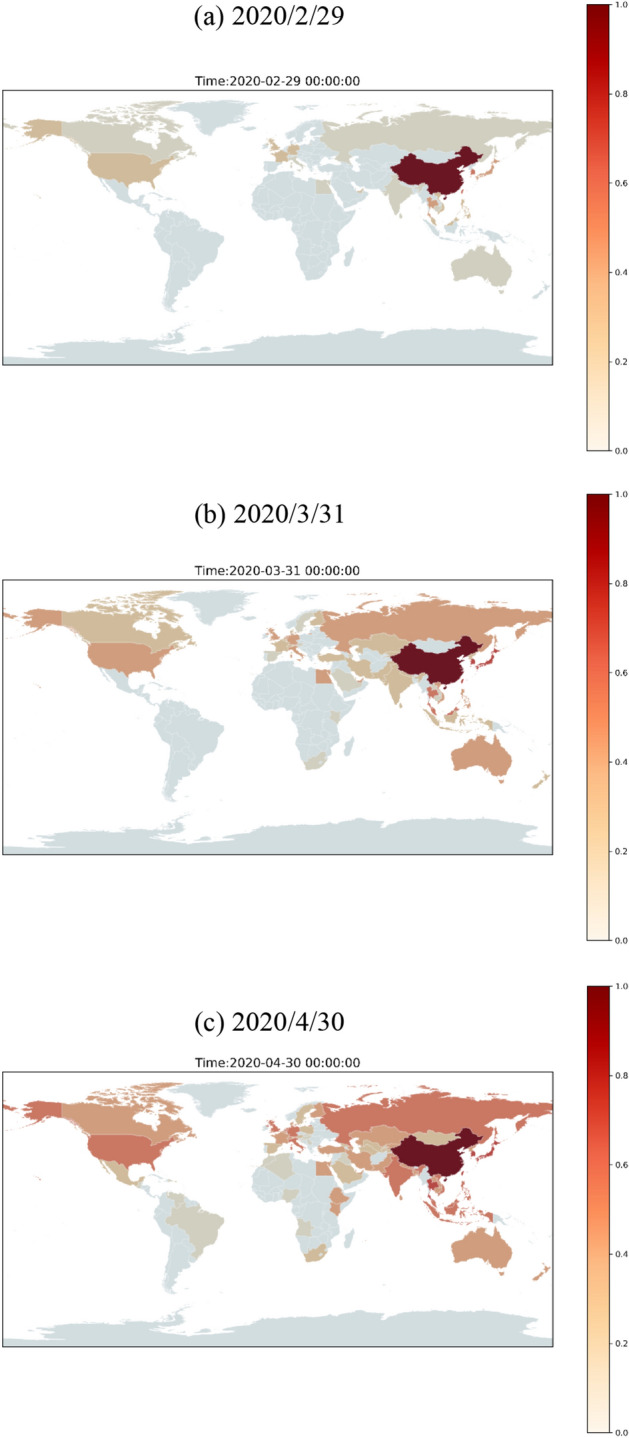
A dynamic risk map for scenario C2 at different time slices (a) 2020/2/29, (b) 2020/3/31, and (c) 2020/4/30. The maps is generated by YCL in Python 3.7 (https://www.python.org/) through the development environment Anaconda 5.3.0 (https://www.anaconda.com/).

Then, the risk calculation method combines hazard with vulnerability and population density to calculate changes over time in the different scenarios of different countries around the world over time to generate the COVID-19 dynamic risk map, as shown in Fig. 9. Here, we show some representative time slices only with scenario C2. For the full time period 2020/1/21–2020/5/31 in the GIF animation format, please refer to Supplementary Materials. Due to each country’s different vulnerability and population density, the spatial distribution will differ slightly from the hazard maps show in Figs. 7 and 8. Some countries have higher risks because of their higher vulnerability or population density. In particular, Asian countries near China have higher risk, particularly East and Southeast Asia. Some other countries have gradually increased their risks over time. For example, countries in Europe, Africa, and South America started increasing their risks from March to April.

## Discussion

This study proposes a framework for the dynamic risk analysis of novel coronavirus SARS-CoV-2-infected pneumonia disease (COVID-19), attempts to understand the impact of vulnerability, and uses this information to simulate the development of the number of potential cases in different countries in the future. In addition, this study compares and quantifies the initial epidemic prevention capabilities and various countries’ management strategies. At present, this study focuses on the spread of risk in space and time, which can be used as early-stage control when a novel infectious disease breaks out. In the future, it should be possible to simulate the changes in the number of recoveries or deaths at the same time to better understand the future risk reduction process. Based on this framework, we can perform the next stage of simulation, continuously modify the risk map, and dynamically update the analysis results. However, there is currently no complete understanding of the characteristics of COVID-19 around the world. There is still a great deal of uncertainty regarding when the epidemic will reach its peak and it is unclear whether recovered patients are still infectious.

Although the outbreak occurred in China, the epidemic is still in its initial stage for the whole world. This study is a preliminary estimation and it can be assumed that COVID-19 is not controlled by climatic factors or effective drugs. Moreover, many cases with mild symptoms or even asymptomatic cases have appeared clinically, which has made epidemic prevention and modeling very difficult. According to this study’s simulation results, if there is no specific medicine, it will likely form a global pandemic.

Although in this study, we collected the proportion of evacuation charter infection cases in various countries and estimated that the total number of cases in Hubei Province is much larger than the actual official case number, we still adopt a more conservative method that uses the official case number for risk assessment. This is based on the assumption that countries are well-prepared for epidemic prevention. We believe that countries have been prepared and have relevant experience for reference earlier than the period when the Wuhan outbreak started. In the next stage, based on the research framework, we can further superimpose the next outbreak on the global impact such as the localized infection that happened in Singapore, and so on.

The other results of many statistical models will be greatly affected by adjusting the parameters. At the start of an unknown infectious disease outbreak, it is impossible to understand the actual parameters. For example, we have been unable to determine the basic reproductive number, serial interval, etc. These will affect the model’s prediction results and create difficulties in model prediction. In fact, we have also tried to use a data-driven approach to make estimations using machine-learning or deep-learning methods such as Long Short-Term Memory (LSTM) and the Random Forest method. However, the preliminary results are poor due to insufficient data and cannot be effectively used for supervised learning.

This study was completed on February 12, 2020. We successfully simulated and predicted the outbreak risks of South Korea, Japan, and Italy in the early stages of the global pandemic, but on March 3, 2020, the outbreak occurred earlier and more severely than expected (risks signal exist in the map as of March 31, 2020 in each scenario of both Figs. 7 and 8). The development of the epidemic is now half a month to a month earlier than we expected, but the trend is similar to our estimation. The localized outbreaks in Japan, South Korea, and Singapore had taken the lead, consistent with the vulnerability analysis and risk analysis results in this study. Japan, South Korea, and Singapore were among the countries with the highest risk in the simulation results. Overall, Taiwan, South Korea, Hong Kong, and Japan are the most vulnerable areas. However, due to the proactive epidemic prevention measures of the Taiwan government and people, the epidemic has been under well control until now. The first case also occurred in Egypt (risks signal exist in the Egyptian part of the map as of March 31, 2020 in each scenario of Figs. 7 and 8). This is consistent with the estimation results in this study. This method can be used as a preliminary risk assessment of the spatiotemporal spread for a new global epidemic in the future.

## Methods

- Data

The original laboratory-confirmed cases data is collected from the WHO situation reports of COVID-19 (WHO, 2020) and the Center for Systems Science and Engineering (CSSE) at Johns Hopkins University^10^ for 2020/1/21–2020/2/12. To define connectivity between countries, international mobility data is calculated from flight route numbers between airports. If more direct routes are found between two airports, this represents higher connectivity. The original route data is accessible from Openflights.org (https://openflights.org/data.html), which is an open-source project to collect and visualize flight information. The world’s Health Care Index is collected from Numbeo (https://www.numbeo.com/health-care/rankings_by_country.jsp), which is the world’s largest database of user-contributed data about cities and countries worldwide. Population density data were collected from the World Bank (https://data.worldbank.org/indicator/EN.POP.DNST). Missing data is interpolated.

b. Two-stage simulation modeling framework
  First stage: imported cases

Assuming that all countries are fully committed to epidemic prevention and well-prepared for epidemic prevention but that there are no specific medicines or vaccines, it is assumed that COVID-19 is not affected by climatic factors (such as temperature). The changes in the case number for each country can be divided into two major stages. The first stage is mainly based on imported cases of immigration from abroad or sporadic cases of local traceable infection sources. The time that the first case happens in each country and the daily number of subsequent cases that continue to occur are based on the country’s Health Care Index and connectivity to the country of outbreak. Here, the country’s vulnerability to outbreak countries for coronavirus is defined as:

2 $$ V_{k} { } = { }\mathop \sum \limits_{i} \frac{{\left( {connectivity} \right)_{ki} }}{{\left( {Health\; Care\; Index} \right)_{k} }} $$

where V is the vulnerability of country *k*, *i* is the outbreak countries, the connectivity is defined as the total number of flight routes between country* k* and country *i*, the outbreak countries. The final vulnerability value is standardized to 0–1 based on the min–max scaling method:

3 $$ x^{\prime} = \frac{{x - x_{min} }}{{x_{max} - x_{min} }} $$

where $x$ is an original value, ${x}^{^{\prime}}$ is the normalized value.

The higher the Health Care Index, the better the resistance against outbreaks from outbreak countries so we have less vulnerability. Meanwhile, higher connectivity indicates that a country has more interactions and higher human mobility with outbreak countries, which makes the disease more easily transmitted to this country, so it has a higher vulnerability.

We determine the time after the first case in each country based on the weight calculation from the vulnerability. In other words, we use the 14-day potential incubation period as a conservative estimate, and determine the time of the first case in inverse proportion to vulnerability. The country with the highest vulnerability will have the first imported case within 14 days, and so on. In the first stage, the daily confirmed cases number is determined by a random number based on the historical data distribution of the daily confirmed cases number and vulnerability in each country. The occurrence of imported cases will be a random process under the influence of the vulnerability between countries. In other words, if a country has a high number of confirmed cases during the first stage, it means that the country either lacks adequate medical conditions and epidemic control measures or is highly vulnerable to China. It is very likely that there will be a higher chance of a confirmed case in the future. In our results section, we estimate and calibrate the vulnerability and the number of confirmed cases for each country.

According to the available data (before February 12, 2020) and examples of local cluster infections in Singapore, > 40 cases are confirmed, which means that the potential for cluster infections is quite high. After entering community cluster infections, the influence of each country's own populace is greater than that of importation. Therefore, it is defined as entering the second stage of simulation after the cumulative case number is > 40 cases. This study was completed before the occurrence of the global pandemic on February 12, 2020. At that time, it was based on the limited initial data to carry out the statistical analysis of the experience threshold, we have found that the local infection cases have accumulated to about 40 cases on average in various countries, and domestic infection cases have begun to appear. At the early stage of the COVID-19 outbreak, COVID-19 is a new disease and full of uncertainty. We have no way to set this number by referring to other infectious diseases. We can only analyze it from the experience gained from actual data. In addition, so far there are no 40 cases from the same flight, especially in the early stages of the global outbreak.

- Second stage: localized outbreaks

It is assumed that in the second stage—after entering the local cluster infection—the pattern change in the number of cases will be similar to that of Hubei Province or other provinces in China. Since this study assumes that future information is clearer than when China started its outbreak, all countries have already prepared to prevent epidemic outbreaks. Therefore, a more conservative method is used to carry out Monte Carlo simulation using data from provinces other than Hubei. Generate 1,000 simulation data of future case growth and extract three scenarios of high (quantile = 0.95), medium (quantile = 0.5), and low (quantile = 0.05) infection from the simulation results while retaining the potential for changes in the number of cases in Hubei as the worst future scenario (Extreme Scenario). Both a sigmoid function and polynomial regression are used to extend the simulation time for different assumed second scenarios, A sigmoid function is used to assume that the epidemic in China has almost reached its peak as a more conservative scenario; polynomial regression is used for the assumption that the epidemic in China will continue and shows no signs of easing (Table 3).

**Table 3:** The setting of simulation scenarios.

First scenarios	Second scenarios	
	1. Conservative	2. Severe
A. Extreme	A1: Extreme–conservative	A2: Extreme–severe
B. High (quantile = 0.95)	B1: High–conservative	B2: High–severe
C. Medium (quantile = 0.5)	C1: Medium–conservative	C2: Medium–severe
D. Low (quantile = 0.05)	D1: Low–conservative	D2: Low–severe

Monte Carlo simulation has been widely used in risk assessment applications with uncertainty in many fields^11–15^. It can perform simulation based on limited data and different scenarios to understand the possible risks since computers can easily simulate a huge number of experimental trials that have random outcomes and uncertainty^16^. The elements of the Monte Carlo simulation method including the expectation ${E}_{\pi }\{U(X)\}$, which is with respect to the probability density π, the response function *U(x)*, and random draws *X* = *x(j)* from the target distribution π^17^.

c. Risk assessment

Based on the Assessment Report published by the IPCC^18–20^. The risk assessment is based on the results of the interaction of various components: hazard, vulnerability, and exposure. In other words, the risk of adverse effects from the impact of extreme events is defined as a function of the vulnerability, exposure, and hazard. Here, extreme events would be outbreaks of COVID-19 in global countries. Among them, we adapt the definition of vulnerability from IPCC; vulnerability is the degree to which a system is susceptible to and unable to cope with the adverse effects of climate change, including climate variability and extremes^20^.

Therefore, according to the most commonly used approach to risk definition, the risk is defined here as:

4 $$ {\text{Risk}} = {\text{ Hazard }} \times {\text{ Exposure }} \times {\text{ Vulnerability}} $$

The infectivity and mortality of COVID-19 under different climatic conditions remain unknown at present, so it is assumed that the infectivity of COVID-19 and the hazard to the human body are the same in all countries. The degree of hazard in each country becomes the estimated number of cases within the country; the greater the number of cases, the greater the potential hazard. Exposure is each country’s population density. We assume that countries with higher population densities are more likely to be exposed to COVID-19. Under the condition that local cluster infection has occurred in the second stage, the higher the population density, the more likely a serious outbreak and thus higher overall risk. Vulnerability is defined as the ratio of connectivity to the Health Care Index calculated in this study, as previously described. At present, only countries’ vulnerability to China is considered. In the future, it can be summed according to whichever countries have experienced large-scale outbreaks based on the superposition principle.

## Supplementary information

Supplementary Information.

## Data Availability

Data are available in a public, open access repository as described in method section.
