# Supplementary material for: The spatiotemporal estimation of the risk and the international transmission of COVID-19: a global perspective
Source: Sci Rep. 2020 Nov 18;10:20021. doi: 10.1038/s41598-020-77242-4 (PMC7676241; doi:10.1038/s41598-020-77242-4)
Supplement: Supplementary file 1 — Supplementary Information. [file 41598_2020_77242_MOESM1_ESM.pptx]

## Slide 1
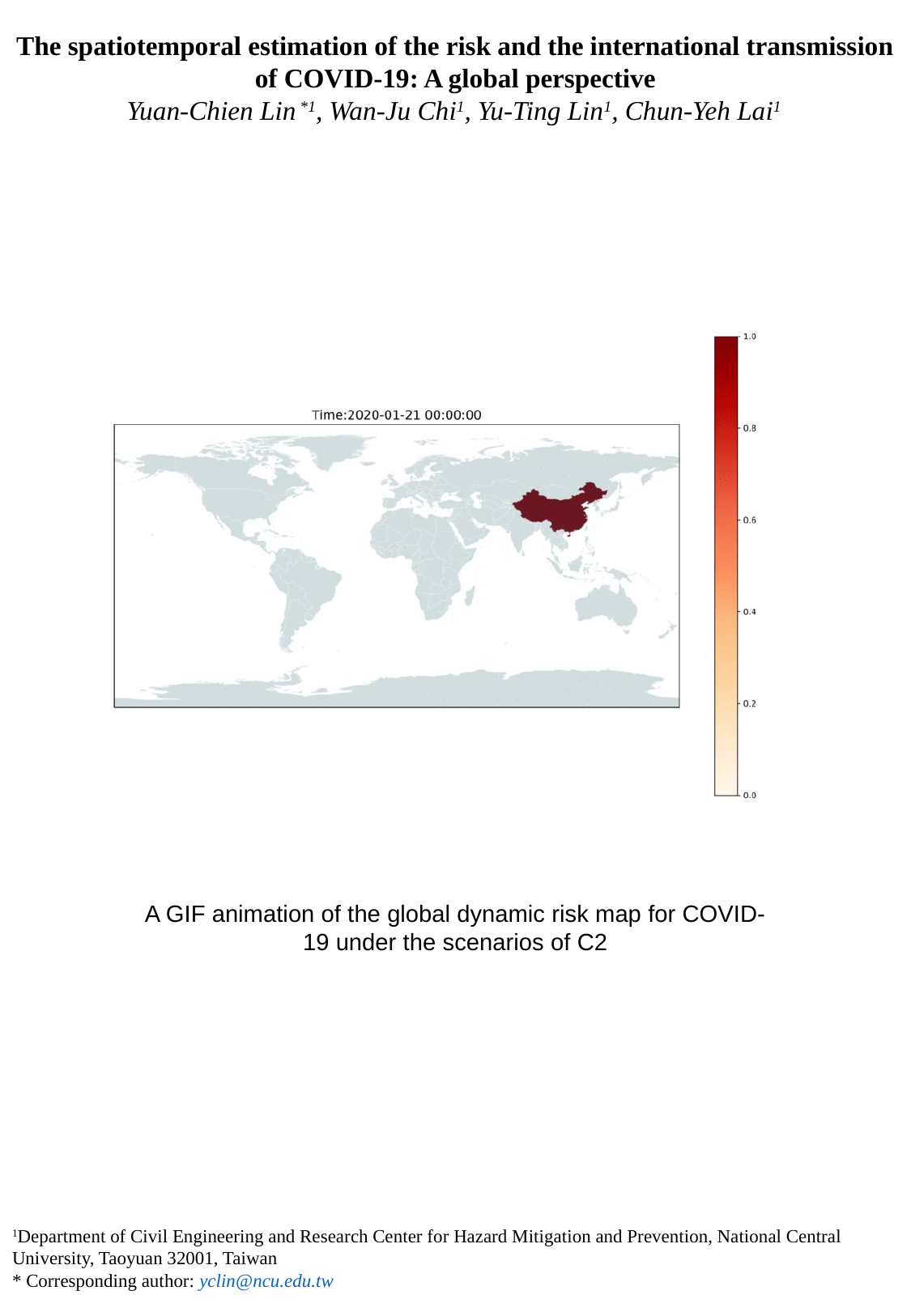

The spatiotemporal estimation of the risk and the international transmission of COVID-19: A global perspective
Yuan-Chien Lin *1, Wan-Ju Chi1, Yu-Ting Lin1, Chun-Yeh Lai1
1Department of Civil Engineering and Research Center for Hazard Mitigation and Prevention, National Central University, Taoyuan 32001, Taiwan
* Corresponding author: yclin@ncu.edu.tw
A GIF animation of the global dynamic risk map for COVID-19 under the scenarios of C2
